# Prescribing Characteristics Associated With Opioid Overdose Following Buprenorphine Taper

**DOI:** 10.1001/jamanetworkopen.2022.34168

**Published:** 2022-09-29

**Authors:** Nikki Bozinoff, Siyu Men, Paul Kurdyak, Peter Selby, Tara Gomes

**Affiliations:** 1 Campbell Family Mental Health Research Institute, Centre for Addiction and Mental Health, Toronto, Ontario, Canada; 2 Department of Family and Community Medicine, University of Toronto, Toronto, Ontario, Canada; 3 ICES, Toronto, Ontario, Canada; 4 Institute for Mental Health Policy Research, Centre for Addiction and Mental Health, Toronto, Ontario, Canada; 5 Department of Psychiatry, University of Toronto, Toronto, Ontario, Canada; 6 Dalla Lana School of Public Health, University of Toronto, Toronto, Ontario, Canada; 7 Li Ka Shing Knowledge Institute, St Michael’s Hospital, Toronto, Ontario, Canada; 8 Leslie Dan Faculty of Pharmacy, University of Toronto, Toronto, Ontario

## Abstract

**Importance:**

Retention in buprenorphine therapy is associated with a lower risk of opioid overdose. Nevertheless, many patients discontinue treatment, and there is limited evidence to guide buprenorphine tapering.

**Objective:**

To understand what prescribing characteristics are associated with opioid overdose following buprenorphine taper.

**Design, Setting, and Participants:**

This is a population-based, retrospective, cohort study of adults who were maintained on buprenorphine for at least 60 days and underwent a buprenorphine taper. The study was conducted in the Canadian province of Ontario, using linked administrative health data. New buprenorphine treatment episodes were accrued between January 1, 2013, and January 1, 2019, and the maximum follow-up was April 30, 2020. Data analysis was performed from December 2020 to August 2022.

**Exposures:**

The primary exposure of interest was time to taper initiation (≤1 year vs >1 year). Secondary exposures included mean rate of taper, percentage days during which the dose was decreasing, and taper duration.

**Main Outcomes and Measures:**

The primary outcome measure was time to fatal or nonfatal opioid overdose within 18 months following treatment discontinuation.

**Results:**

Among 5774 individuals, the median (IQR) age at index date was 34 (28-44) years, and 3462 individuals (60.0%) were male. Time to taper initiation longer than 1 year vs 1 year or less (6.73 vs 10.35 overdoses per 100 person-years; adjusted hazard ratio [aHR], 0.69; 95% CI, 0.48-0.997), a lower mean rate of taper (≤2 mg per month, 6.95 overdoses per 100 person-years; >2 to ≤4 mg per month, 11.48 overdoses per 100 person-years; >4 mg per month, 17.27 overdoses per 100 person-years; ≤2 mg per month vs >4 mg per month, aHR, 0.65; 95% CI, 0.46-0.91; >2 to ≤4 mg per month vs >4 mg per month, aHR, 0.69; 95% CI, 0.51-0.93), and dose decreases in 1.75% or less of days vs more than 3.50% of days during the taper period (5.87 vs 13.87 overdoses per 100 person-years; aHR, 0.64; 95% CI, 0.43-0.93) were associated with reduced risk of opioid overdose; however, taper duration was not.

**Conclusions and Relevance:**

In this retrospective cohort study, buprenorphine tapers undertaken after at least 1 year of therapy, a slower rate of taper, and a lower percentage of days during which the dose was decreasing were associated with a significantly lower risk of opioid overdose, regardless of taper duration. These findings underscore the importance of a carefully planned taper and could contribute to reduction in opioid-related overdose death.

## Key Points

**Question:**

Among people receiving buprenorphine maintenance therapy and undergoing a taper, what prescribing characteristics are associated with opioid overdose?

**Findings:**

In this cohort study of 5774 individuals undergoing a buprenorphine taper, a longer time to taper initiation (≥1 year vs <1 year), a lower mean rate of taper (≤2 mg per month and >2 to ≤4 mg per month vs >4 mg per month), and a lower percentage of days during which the dose was decreasing (≤1.75% vs >3.50% of taper days) were significantly associated with a reduced risk of opioid overdose. Taper duration was not significantly associated with overdose.

**Meaning:**

Buprenorphine tapers undertaken after at least 1 year of therapy, those with a slower rate of taper, and a lower percentage of days during which the dose was decreasing were associated with a significantly lower risk of opioid overdose, regardless of taper duration.

## Teaser

This cohort study examines what prescribing characteristics are associated with opioid overdose following buprenorphine taper.

## Introduction

North America is facing a devastating opioid overdose crisis, with more than 6300 opioid overdose deaths in Canada in 2020 and more than 90 000 in the US in the same year.^1,2^ Medications for opioid use disorder (MOUD), buprenorphine and methadone, are a key response to the crisis and are associated with a reduced risk of mortality.^3,4^

Although most clinical guidelines recommend that MOUD be continued indefinitely,^5,6^ retention in MOUD is low.^7,8,9^ Tapering MOUD is inferior to maintenance therapy because of higher rates of relapse to illicit opioids among those who discontinue treatment^10,11^ and subsequent overdose death.^12,13,14^ Despite these risks, clients report not wanting to be dependent on a stigmatized medication, loss of freedom, adverse effects, and high costs as motivations for discontinuation.^7,8,9,15,16,17,18^ Involuntary discontinuation from MOUD has also been described and sometimes occurs as a result of insurance policies with time limits on coverage, in residential treatment settings requiring abstinence, or as a disciplinary measure.^19,20^

A few studies^21,22,23,24,25,26,27,28^ have considered prescribing characteristics associated with successful methadone tapering. In some observational studies,^21,22,28^ clients with longer treatment duration before taper had a higher likelihood of abstinence, whereas in other studies^26,29^ they did not. A population-based retrospective study^29^ from British Columbia found that methadone tapers lasting longer than 52 weeks, regardless of how early in the treatment episode the taper was initiated, and a slower and more gradual tapering schedule provided the highest odds of sustained abstinence. There is similarly limited evidence to guide buprenorphine tapering, and the American Society of Addiction Medicine OUD treatment guideline notes that further research is needed in this area.^30^

Buprenorphine is now the first-line treatment for OUD,^5^ and its use continues to increase.^31,32^ Considering the risks associated with discontinuation of buprenorphine, the objective of this study was to identify prescribing characteristics associated with opioid overdose and return to opioid use following buprenorphine taper. Understanding how to taper buprenorphine safely is an important, client-centered question that could have major implications for OUD treatment.

## Methods

### Study Design and Setting

We conducted a population-based, retrospective cohort study using linked administrative health data from the province of Ontario, Canada. Approximately 14 million people reside in Ontario and receive publicly insured health care coverage by a single payer, the government of Ontario. The use of data in this project was authorized under section 45 of Ontario’s Personal Health Information Protection Act, which did not require review by a research ethics board. The reporting of this study followed the Strengthening the Reporting of Observational Studies in Epidemiology (STROBE) reporting guideline.

### Data Sources

All data for this study were held at ICES, an independent, nonprofit research institute whose legal status under Ontario’s health information privacy law allows it to collect and analyze health care and demographic data, without consent, for health system evaluation and improvement. The main data sources used were the Narcotics Monitoring System (NMS) and the Drug and Drug-Alcohol Related Death database. NMS is a mandatory prescription reporting system in Ontario that captures all outpatient prescriptions for opioids, benzodiazepines, and stimulants across Ontario, regardless of payment method.^33^ The Drug and Drug-Alcohol Related Death database is a data set of confirmed opioid-related deaths from the Office of the Chief Coroner of Ontario.^34^ More details on administrative health data databases and codes used can be found in eTable 1 and eTable 2 in the Supplement. These data sets were linked using unique encoded identifiers.

### Cohort Definition

We identified a cohort of Ontario residents, aged 18 years or older, with at least 1 buprenorphine treatment episode between January 1, 2013, and January 1, 2019, inclusive, who subsequently discontinued treatment. A new treatment episode was defined as continuous buprenorphine treatment for a period of at least 60 days with prescription interruptions of no more than 13 consecutive days, and no buprenorphine use in the 30 days before accrual (eFigure 1 in the Supplement). A minimum period of continuous use of 60 days was chosen because short-term therapy results in greater than 90% risk of return to opioid use,^11,35^ and we were interested in understanding tapering following maintenance therapy. In Ontario, buprenorphine dispensing is highly regimented, and daily dose was calculated on the basis of prescription records (eAppendix in the Supplement). We excluded individuals with missing patient identifiers, data on age or sex, those younger than 18 or older than 105 years, those who were not Ontario residents, and those whose buprenorphine dose never went above 2 mg. To restrict the cohort to episodes that involved a taper, only those episodes where the daily dose was 2 mg or less in the final dispense of the treatment episode or where the dose at discontinuation was less than the dose 4 weeks earlier (ie, dose is decreasing in the last 4 weeks of treatment) were included^29^ (eFigure 2 in the Supplement).

Discontinuation was defined as a gap in buprenorphine prescriptions of at least 14 consecutive days. There is substantial variation in the literature in defining MOUD discontinuation. Sixty days,^36,37^ 2 times the days supplied,^38^ 6 days,^39^ 10 days,^40^ and 14 days^41^ have been used previously. We used 14 days to describe discontinuation because among those undergoing a taper who discontinue therapy and feel discomfort, return to MOUD may be rapid. Our definition, therefore, describes a clinically meaningful break in therapy that would require a buprenorphine reinitiation, while also allowing us to capture return to buprenorphine following an unsuccessful taper attempt as an outcome.

### Exposure Definitions

The primary exposure of interest was time to taper initiation, defined as the time from treatment initiation to the onset of the tapering period and dichotomized into 1 year or less vs longer than 1 year. The taper start date was defined by looking for the highest buprenorphine daily dose after day 42 (to avoid misclassifying overshooting of the maintenance dose, which often occurs within the first 6 weeks of therapy) that lasted for at least 7 consecutive days, and then choosing the chronological latest date of that dose. We also defined several secondary exposures of interest. First, we calculated the mean number of milligrams decreased per 4 weeks (ie, taper rate per month), categorized into 2 mg or less per month, more than 2 mg to 4 mg or less per month, and more than 4 mg per month. Second, we determined the percentage of days during which the dose was decreasing, defined as the total number of dose decreases during the taper episode, divided by the total duration of taper (days), and categorized as less than or equal to 1.75%, more than 1.75% to less than or equal to 3.50%, and more than 3.50% of days decreasing during the taper period. This type of variable has been used previously in the methadone literature^29^ and is a measure of optimal frequency of scheduled dose decreases. A lower percentage of days during which the dose was decreasing corresponds to greater time between dose decreases. For example, dose decreases on less than or equal to 1.75% of days corresponds roughly to dose decreases every 2 months or less often for tapers lasting at least 2 months. Finally, taper duration was defined as the time from the start of buprenorphine taper to end of the treatment episode (measured in days) and categorized as 6 months or less, more than 6 to less than or equal to 12 months, and more than 1 year. In all cases, categories were determined to align with clinical practice and to support clinical interpretation of results.

### Outcomes

For the primary outcome (opioid overdose), individuals were followed forward from buprenorphine discontinuation date (date of last dispensed claim) to the outcome of interest, end of data availability (April 30, 2020), or 548 days (18 months), whichever came first. The primary outcome was time to opioid overdose, defined as any emergency department visit or hospitalization for opioid overdose based on *International Statistical Classification of Diseases and Related Health Problems, Tenth Revision* codes (eTable 2 in the Supplement) or any opioid-related death as determined by an investigating coroner^34^ and censoring on non–opioid-related death, rotation to methadone within 14 days of discontinuation, and MOUD treatment reentry. Secondary outcome measures included MOUD treatment reentry, defined as any return to methadone or buprenorphine, censoring on all-cause mortality; and prescription opioid use, defined as any non-MOUD opioid prescriptions of duration greater than 14 days, censoring on all-cause mortality and return to MOUD. For the MOUD treatment reentry analysis, the follow-up had to be shifted 14 days because, by definition, individuals had to have no MOUD prescriptions within 14 days of episode completion to be considered discontinued. For this analysis, therefore, persons who rotated to methadone within 14 days of episode completion or who died within 14 days of episode completion were excluded.

### Statistical Analysis

Data analysis was performed from December 2020 to August 2022. For the purpose of the primary analyses, we included only the first episode for each individual. The analyses were completed using SAS statistical software version 9.3 (SAS Institute), and 2-tailed *P* < .05 was deemed significant. There was missingness in only 2 variables (income quintile and rurality), and these were grouped separately. We described the baseline characteristics of the cohort stratified by the primary exposure variable and compared groups using standardized differences. A standardized difference greater than 0.10 was considered meaningful.^42^ Kaplan-Meier curves were used to present crude survival for each outcome, and the log-rank test was used to determine whether survival functions between each strata were significantly different.

We used multivariable Cox proportional hazards regression modeling to characterize the association between the exposure variables and our primary outcome, while adjusting for important confounders. Covariates included in multivariable Cox models to control for confounding were determined a priori according to clinical expertise and their known or hypothesized association with the exposure and outcome. They included age, sex, income quintile, rurality, comorbidity burden measured using The Johns Hopkins ACG System version 10,^43^ harmful sedative-hypnotic use or dependence, harmful stimulant use or dependence, harmful alcohol use or dependence, hospitalization or ED visit for depression in last 2 years, hospitalization or ED visit for anxiety in last 2 years, history of opioid overdose in last 1 year, having a pain prescriber at baseline, history of methadone in last 6 months, history of buprenorphine in the last 6 months, maximum dose during the treatment episode, the percentage of missed doses, and calendar year of buprenorphine discontinuation (2013-2015 vs 2016 or later) to control for the prefentanyl and fentanyl era (eTable 2 in the Supplement). When modeling the secondary outcomes, we adjusted for the same confounders described already.

We tested the proportional hazards assumption by adding time-varying covariates for our exposure variables in multivariable models, as well as by visually inspecting the martingale residuals. If models failed either of these assessments, nonproportionality was assumed and we added time-varying covariates to the model and reported hazard ratios (HRs) at different time points over the follow-up period.

Multiple sensitivity analyses were conducted. First, we included all treatment episodes that met our inclusion criteria to increase our cohort size. To account for dependence between multiple episodes per individual, we used an Andersen-Gill recurrent events model.^44^ Second, to understand whether optimal taper characteristics were simply a proxy for people who were more likely to follow physician advice and might do well regardless of taper characteristics, we conducted a sensitivity analysis among persons who successfully completed a taper (ie, those whose last filled prescription was ≤2 mg). Finally, we conducted a post hoc sensitivity analysis using a subdistribution hazards (SH) model because cause-specific hazards models may overestimate hazards in the setting of a large number of competing events.^45,46^

## Results

We included 5774 first episodes in the primary analysis and an additional 677 recurrent episodes for a total of 6451 episodes in the recurrent event analysis (Figure). Baseline characteristics of the primary cohort of 5774 individuals, stratified by time to taper initiation, are described in Table 1. The median (IQR) age at index was 34 (28-44) years, and 3462 participants (60.0%) were male. The population was mostly urban (4852 individuals [84.0%]) and of low socioeconomic status (3296 individuals [57.1%] were in the first and second income quintiles). In total, 759 individuals (13.2%) had a hospital visit for nonfatal opioid overdose in the year before the index date. Baseline characteristics were similar among those with a time to taper initiation less than or equal to 1 year compared with more than 1 year. Those with a longer time to taper initiation were slightly older at the time of buprenorphine initiation and had a lower prevalence of ED visits or hospitalization for depression and anxiety (Table 1). The median (IQR) time to taper initiation was 122 (72-249) days (eTable 3 in the Supplement).

**Figure.**
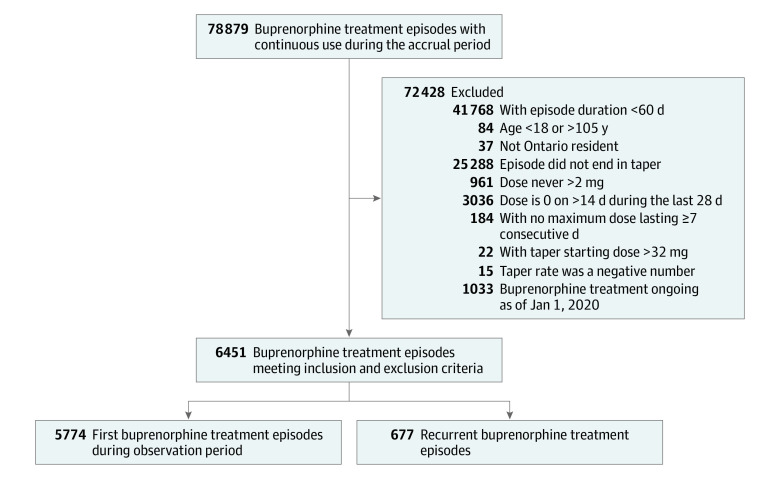
Cohort Assembly Flowchart

**Table 1.** Baseline Characteristics Stratified by Time to Taper Initiation

Variable	Participants, No. (%)		
	Total	≤1 y to taper initiation	>1 y to taper initiation
Participants, No. (%)	5774 (100.0)	4954 (85.8)	820 (14.2)
Age, median (IQR), y	34 (28-44)	34 (27-44)	35 (29-46)^a^
Sex			
Female	2312 (40.0)	1977 (39.9)	335 (40.9)
Male	3462 (60.0)	2977 (60.1)	485 (59.1)
Rurality			
Urban	4852 (84.0)	4163 (84.0)	698 (84.0)
Rural	891 (15.4)	764 (15.4)	127 (15.5)
Missing	31 (0.5)	27 (0.5)	4 (0.5)
Income quintile			
First (lowest)	2085 (36.1)	1786 (36.1)	299 (36.5)
Second	1211 (21.0)	1039 (21.0)	172 (21.0)
Third	942 (16.3)	807 (16.3)	135 (16.5)
Fourth	766 (13.3)	646 (13.0)	120 (14.6)
Fifth (highest)	730 (12.6)	640 (12.9)	90 (11.0)
Missing	40 (0.7)	36 (0.7)	≤5 (0.5)
Hospitalization or ED visit for depression in last 2 y	100 (1.7)	95-99^b^^,^^c^	≤5 (0.5)^a^^,^^b^
Hospitalization or ED visit for anxiety in last 2 y	121 (2.1)	115 (2.3)	6 (0.7)^a^
Concurrent benzodiazepine prescription	930 (16.1)	784 (15.8)	146 (17.8)
Substance abuse			
Alcohol use or dependence	674 (11.7)	575 (11.6)	99 (12.1)
Stimulant use or dependence	195 (3.4)	174 (3.5)	21 (2.6)
Sedative-hypnotic use or dependence	98 (1.7)	82 (1.7)	16 (2.0)
Hospitalization or ED visit for opioid overdose in last 1 y	759 (13.2)	664 (13.4)	95 (11.6)
Buprenorphine use in last 6 mo	1452 (25.2)	1248 (25.2)	204 (24.9)
Methadone use in last 6 mo	1381 (23.9)	1204 (24.3)	177 (21.6)
Prescription stimulant use in last 6 mo	353 (6.1)	311 (6.3)	42 (5.1)
Prescription benzodiazepine use in last 6 mo	1576 (27.3)	1322 (26.7)	254 (31.0)
Prescription opioid use (excluding medications to treat opioid use disorder) in last 6 mo	1860 (32.2)	1583 (32.0)	277 (33.8)
Adjusted diagnostic groups, median (IQR)	6 (4-10)	6 (4-10)	7 (4-10)
Chronic obstructive pulmonary disease	521 (9.0)	441 (8.9)	80 (9.8)
Diabetes	354 (6.1)	303 (6.1)	51 (6.2)
HIV	40 (0.7)	33 (0.7)	7 (0.9)
Traumatic brain injury	334 (5.8)	291 (5.9)	43 (5.2)
Pain prescriber	471 (8.2)	402 (8.1)	69 (8.4)
Physician visits last 1 y, median (IQR), No.	10 (3-22)	10 (3-22)	11 (4-23)
ED visits in the last 1 y			
0	2467 (42.7)	2100 (42.4)	367 (44.8)
1	1212 (21.0)	1048 (21.2)	164 (20.0)
≥2	2095 (36.3)	1806 (36.5)	289 (35.2)

Abbreviation: ED, emergency department.

^a^ Denotes a standardized difference greater than 0.1.

^b^ Institutional policy requires suppression of cells with 5 or fewer individuals.

^c^ Data in this cell are shown as a range and without a percentage to prevent back-calculation of the value in the suppressed cell.

In this cohort of 5774 individuals undergoing a buprenorphine taper, 349 individuals experienced an opioid overdose (9.56 overdoses per 100 person-years), 3360 reinitiated MOUD (96.41 events per 100 person-years), and 463 started prescription opioids (13.88 events per 100 person-years); 292 individuals rotated to methadone within 14 days of buprenorphine discontinuation. Overall, 3799 individuals (66.0%) experienced at least 1 of the outcome measures (any opioid overdose, MOUD reentry, or prescription opioid use) within 18 months following buprenorphine taper (118.5 events per 100 person-years).

Kaplan-Meier curves are presented in eFigures 3 to 14 in the Supplement. In multivariable models, a longer time to taper initiation (≥1 year vs <1 year) was associated with a significantly lower risk of opioid overdose (6.73 vs 10.35 overdoses per 100 person-years; adjusted HR [aHR], 0.69; 95% CI, 0.48-0.997). Similarly, lower mean rates of taper were associated with reduced risk of opioid overdose compared with higher rates of taper (≤2 mg per month, 6.95 overdoses per 100 person-years; >2 to ≤4 mg per month, 11.48 overdoses per 100 person-years; >4 mg per month, 17.27 overdoses per 100 person-years; ≤2 mg per month vs >4 mg per month, aHR, 0.65; 95% CI, 0.46-0.91; >2 to ≤4 mg per month vs >4 mg per month, aHR, 0.69; 95% CI, 0.51-0.93). A lower percentage of days during which the dose was decreasing was also associated with reduced risk of opioid overdose compared with a higher percentage of treatment days with a decreasing dose (≤1.75% vs >3.50% of days, 5.87 vs 13.87 overdoses per 100 person-years; aHR, 0.64; 95% CI, 0.43-0.93); however, taper duration was not significantly associated with overdose (Table 2).

**Table 2.** Multivariable Cox Proportional Hazards Model of Buprenorphine Taper Characteristics Associated With Opioid Overdose Within 18 Months After Discontinuation

Variable	Total individuals, No. (N = 5774)	Individuals with opioid overdose, No.	Overdose rate per 100 person-years	Adjusted HR (95% CI)^a^
Time to taper initiation, y				
≤1	4954	315	10.35	1.00 [Reference]
>1	820	34	6.73	0.69 (0.48-0.997)
Taper rate, milligrams per month				
≤2	3258	154	6.95	0.65 (0.46-0.91)
>2 to ≤4	1026	69	11.48	0.69 (0.51-0.93)
>4	1490	126	17.27	1.00 [Reference]
Percentage of days during which dose was decreasing				
≤1.75	1914	71	5.87	0.64 (0.43-0.95)
>1.75 to ≤3.50	1241	76	8.63	0.84 (0.61-1.16)
>3.50	2619	202	13.87	1.00 [Reference]
Taper duration				
≤6 mo	4024	270	11.70	1.00 [Reference]
>6 to ≤12 mo	898	52	8.10	1.12 (0.79-1.60)
>1 y	852	27	4.52	0.75 (0.47-1.21)

Abbreviation: HR, hazard ratio.

^a^ The proportional hazards assumption was not violated when time-varying covariates were added (*P* = .76), nor when examining the martingale residuals.

In the secondary analyses, tapering at a mean rate of less than or equal to 2 mg per month (compared with >4 mg) was associated with a lower risk of MOUD reentry within 182 days after treatment discontinuation (84.03 vs 125.56 events per 100 person-years; aHR, 0.83; 95% CI, 0.72-0.95), although this was not observed at later follow-up times (eTable 4 in the Supplement and Table 3). A lower percentage of days during which dose was decreasing (≤1.75%) during the taper period was associated with a higher risk of MOUD reentry compared with more than 3.50% days decreasing (96.53 vs 109.79 events per 100 person-years; aHR, 1.30; 95% CI, 1.16-1.46). Longer taper duration (>6 to ≤12 months and >1 year vs ≤6 months) was associated with a lower risk of MOUD reentry, with the effect being more pronounced later at 548 days of follow-up (>6 to ≤12 months, 71.08 events per 100 person-years; >1 year, 72.99 events per 100 person-years; ≤6 months, 109.35 events per 100 person-years; >6 to ≤12 months vs ≤6 months, aHR, 0.52; 95% CI, 0.34-0.80; >1 year vs ≤6 months, aHR, 0.33; 95% CI, 0.20-0.55). Finally, buprenorphine taper characteristics were not associated with time to prescription opioid use, with the exception of the rate of taper (Table 4). Specifically, tapering at a mean rate of 2 mg or less per month (compared with >4 mg per month) was associated with a lower risk of prescription opioid use (10.80 vs 22.30 events per 100 person-years; aHR, 0.61; 95% CI, 0.45-0.83).

**Table 3.** Multivariable Cox Proportional Hazards Model of Buprenorphine Taper Characteristics Associated With Treatment Reentry Within 18 Months After Discontinuation

Characteristic	Adjusted HR (95% CI) (N = 5449)^a^			
	Overall	182 d	365 d	548 d
Time to taper initiation, y				
≤1	1.00 [Reference]	NA	NA	NA
>1	0.96 (0.87-1.07)	NA	NA	NA
Taper rate, milligrams per month^b^				
≤2	NA	0.83 (0.72-0.95)	0.98 (0.77-1.25)	1.16 (0.80-1.68)
>2 to ≤4	NA	0.99 (0.87-1.13)	1.18 (0.90-1.54)	1.40 (0.92-2.12)
>4	NA	1.00 [Reference]	1.00 [Reference]	1.00 [Reference]
Percentage of days during which dose was decreasing				
≤1.75	1.30 (1.16-1.46)	NA	NA	NA
>1.75 to ≤3.5	0.92 (0.82-1.03)	NA	NA	NA
>3.5	1.00 [Reference]	NA	NA	NA
Taper duration				
≤6 mo	NA	1.00 [Reference]	1.00 [Reference]	1.00 [Reference]
>6 to ≤12 mo	NA	0.71 (0.62-0.82)	0.61 (0.46-0.80)	0.52 (0.34-0.80)
>1 y	NA	0.62 (0.52-0.73)	0.45 (0.33-0.63)	0.33 (0.20-0.55)

Abbreviations: HR, hazard ratio; NA, not applicable.

^a^ The number of individuals analyzed is 5449 because the outcome window was shifted 14 days in this analysis to account for the fact that discontinuation was defined as no return to buprenorphine within 14 days.

^b^ The proportional hazards assumption was violated when time-varying covariates were added (*P* = .03) and in examining the martingale residuals, therefore HRs are reported at 3 times over the follow-up period for taper rate and taper duration.

**Table 4.** Multivariable Cox Proportional Hazards Model of Buprenorphine Prescribing Characteristics Associated With Prescription Opioid Use Within 18 Months After Treatment Discontinuation

Variable	Total No. of individuals (N = 5774)	Individuals with prescription opioid use, No.	Prescription opioid use rate per 100 person-years	Adjusted HR (95% CI)^a^
Time to taper initiation				
≤1 y	4954	405	14.11	1.00 [Reference]
>1 y	820	58	12.43	0.81 (0.61-1.09)
Taper rate, milligrams per month				
≤2	3258	227	10.80	0.61 (0.45-0.83)
>2 to ≤4	1026	87	15.37	0.87 (0.67-1.15)
>4	1490	149	22.30	1.00 [Reference]
Percentage of days during which dose was decreasing				
≤1.75	1914	123	10.74	1.08 (0.78-1.49)
>1.75 to ≤3.50	1241	109	13.21	1.25 (0.94-1.66)
>3.50	2619	231	16.92	1.00 [Reference]
Taper duration				
≤6 mo	4024	345	15.96	1.00 [Reference]
>6 to ≤12 mo	898	71	11.82	1.02 (0.76-1.37)
>1 y	852	47	8.19	0.78 (0.54-1.13)

Abbreviation: HR, hazard ratio.

^a^ The proportional hazards assumption was not violated when time-varying covariates were added (*P* = .40), nor when examining the martingale residuals.

### Sensitivity Analyses

The recurrent event analyses for all outcome measures were consistent with findings from the Cox models and can be found in the eTables 5 to 8 in the Supplement. In the sensitivity analysis considering only successful tapers (ie, episodes with an end dose ≤2 mg; 3154 episodes), results were similar to the outcomes from the primary analysis with a lower mean rate of taper and lower percentage days during which the dose was decreasing being associated with reduced risk of opioid overdose and taper duration not significantly associated with overdose (eTable 9 in the Supplement). However, in contrast to the primary analysis, time to taper start less than or equal to 1 year was not significantly associated with opioid overdose. Finally, the results of the subdistribution hazards models were similar to those of the cause-specific models with the exception of rate of taper, which was not significantly associated with opioid overdose (eTables 10 and 11 in the Supplement).

## Discussion

Although it is well documented that discontinuation of MOUD is associated with overdose,^14,47,48^ to our knowledge, this cohort study is the first to associate buprenorphine taper characteristics with opioid overdose risk. Specifically, buprenorphine tapers undertaken after at least 1 year of therapy, those with a slower rate of taper, and more time between dose decreases were associated with a significantly lower risk of opioid overdose, regardless of taper duration.

Across all of our analyses, the taper characteristic that was most consistently associated with reduced risk of return to opioid use was a mean taper rate of 2 mg or less per month over the taper period. The importance of a slow taper is consistent with research in the methadone literature. For example, data from a randomized clinical trial^49^ of clients maintained on a stable dose of methadone for at least 1 year found that gradual tapering (no more than 3% of initial dose per week) resulted in fewer treatment dropouts compared with rapid tapering (10% per week). Similarly, a population-based study^29^ of methadone prescribing characteristics associated with sustained abstinence in British Columbia found that dose decreases of 5% to 15% had higher odds of success compared with dose decreases of more than 90%. It is reasonable, therefore, to recommend slower tapers to patients requesting discontinuation.

Our results also point to the fact that it may be important to understand the optimal duration of different treatment phases rather than overall time spent in MOUD treatment. We found that longer time to taper initiation (≥1 year) was independently associated with a lower risk of opioid overdose compared with initiating a taper in the first year of treatment. Our results are consistent with some observational studies of methadone,^21,22^ which have found a longer treatment duration before taper is associated with a higher likelihood of abstinence. We also found that a longer taper duration was significantly associated with reduced risk of MOUD reentry. Existing US population-based data have suggested that longer total buprenorphine treatment durations are associated with better treatment outcomes.^13,50^ On the basis of our results, time to taper initiation may be more important in relation to risk of opioid overdose, whereas longer duration of taper may be associated with reduced risk of MOUD reentry. Further research is required to understand more fully the optimal length of each phase of treatment for patients who have a goal to discontinue MOUD.

Finally, among this cohort of 5774 individuals undergoing a buprenorphine taper after a period of maintenance therapy, 66.0% experienced at least 1 outcome measure (opioid overdose, MOUD reentry, or prescription opioid use) within 18 months following buprenorphine discontinuation (118.5 events per 100 person-years), suggesting that many people will return to opioid use following buprenorphine taper. This is consistent with observational studies from the early methadone literature, suggesting rates of abstinence between 30% to 35% around 18 to 24 months of follow-up,^21,23^ as well as results from a 2005 review^51^ that found a pooled estimate of post-MOUD abstinence to be 33%. It is important to recognize that taper characteristics are only part of the complex biological, psychological, and social milieu that likely contribute to sustained abstinence following buprenorphine taper.^52^ Stable home life,^7,21^ abstinence from illicit substances on urine drug screening before taper initiation,^17,21^ the recommendation of readiness for taper from program staff,^53^ shorter duration of illicit opioid use,^25,54^ having social supports,^18^ and increased patient motivation^17,26^ have all been associated with sustained abstinence following treatment. Overall, our results highlight the possible risks associated with buprenorphine tapering, including the risk of opioid overdose, and support the idea that treatment providers, regulatory agencies, insurers, and mutual-support groups must work to remove structural barriers and stigma that discourage participation in long-term MOUD. For those clients who do choose to taper, our results provide tangible guidance to patients and clinicians with respect to when and how to do so. Our results also reinforce the importance of informed consent for tapering and pairing any tapering with appropriate harm reduction counselling and services, including ensuring that take-home naloxone is available.

### Strengths and Limitations

The generalizability of our study is strengthened by the use of NMS, which captures all outpatient prescriptions for controlled substances, regardless of payer, across Canada’s most populous province. Our study is, however, subject to a number of limitations. First, we were not able to capture use of illicit opioids where the individual does not have an opioid-related overdose, does not reenter MOUD, or is not prescribed other opioids. This means that some people who return to opioid use will be misclassified as abstinent, and, therefore, the rate of return to opioid use reported (66.0% within 18 months after discontinuation) may be an underestimate. Second, although prescribing records reflect what was dispensed to the patient, we cannot be certain that the patient took buprenorphine as prescribed, in whole or in part. Similarly, assumptions were made with respect to the calculation of daily dose from prescription records (eAppendix in the Supplement). This may result in misclassification of buprenorphine taper characteristics. Third, because we used administrative health data, no inferences with respect to why tapers were initiated can be made. Fourth, as with all observational studies, our study is subject to bias from unmeasured confounding. Social stability and ongoing illicit drug use, for example, cannot be captured in administrative data.

## Conclusions

In this large, population-based cohort of individuals undergoing buprenorphine taper after a maintenance period, starting a taper after at least 1 year of therapy, a slower rate of taper, and a lower percentage of taper days during which the dose was decreasing were all associated with lower risk of opioid overdose. Our results highlight that MOUD must continue to be offered within a maintenance framework—specifically, treatment with buprenorphine should be continued for as long as patients benefit from it and want to continue with it. For patients who do want to discontinue therapy, our results underscore the importance of a carefully planned taper, and have important implications for OUD treatment.

## Notes

Supplement. **eTable 1.** Descriptions of All Linked Administrative Databases Used in the Study **eTable 2.** Covariate Definitions **eFigure 1.** Study Schematic **eAppendix.** Calculation of Daily Dose **eFigure 2.** Cohort Assembly **eTable 3.** Distribution of the Primary Exposure, Time to Taper Initiation **eFigure 3.** Kaplan-Meier Curve of Time to Opioid Overdose Following Buprenorphine Taper, Stratified by Time to Taper Start (*P* = .02) **eFigure 4.** Kaplan-Meier Curve of Time to Opioid Overdose Following Buprenorphine Taper, Stratified by Milligrams Tapered per Month (*P* < .001) **eFigure 5.** Kaplan-Meier Curve of Time to Opioid Overdose Following Buprenorphine Taper, Stratified by Percentage of Days Descending During the Taper Period (*P* < .001) **eFigure 6.** Kaplan-Meier Curve of Time to Opioid Overdose Following Buprenorphine Taper, Stratified by Taper Duration (*P* < .001) **eFigure 7.** Kaplan-Meier Curve of Time to MOUD Reentry Following Buprenorphine Taper, Stratified by Time to Taper Start (*P* = .12) **eFigure 8.** Kaplan-Meier Curve of Time to MOUD Reentry Following Buprenorphine Taper, Stratified by Milligrams Tapered per Month (*P* < .001) **eFigure 9.** Kaplan-Meier Curve of Time to MOUD Reentry Following Buprenorphine Taper, Stratified by Percentage of Days the Dose Was Decreasing During the Taper Period (*P* < .001) **eFigure 10.** Kaplan-Meier Curve of Time to MOUD Reentry Following Buprenorphine Taper, Stratified by Taper Duration (*P* < .001) **eFigure 11.** Kaplan-Meier Curve of Time to Prescription Opioid Use Following Buprenorphine Taper, Stratified by Time to Taper Start (*P* = .34) **eFigure 12.** Kaplan-Meier Curve of Time to Prescription Opioid Use Following Buprenorphine Taper, Stratified by Milligrams Tapered per Month (*P* < .001) **eFigure 13.** Kaplan-Meier Curve of Time to Prescription Opioid Use Following Buprenorphine Taper, Stratified by Percentage of Days the Dose Was Decreasing During the Taper Period (*P* = .001) **eFigure 14.** Kaplan-Meier Curve of Time to Prescription Opioid Use Following Buprenorphine Taper, Stratified by Taper Duration (*P* < .001) **eTable 4.** Event Rates for Buprenorphine Taper Characteristics Associated With Treatment Reentry Within 18 Months Postdiscontinuation (N = 5449) **eTable 5.** Recurrent Event Analysis of Buprenorphine Taper Characteristics Associated With Opioid Overdose Within 18 Months Posttreatment Discontinuation (N = 6451) **eTable 6.** Recurrent Event Analysis of Buprenorphine Taper Characteristics Associated With Treatment Reentry Within 18 Months Postdiscontinuation (N = 6086) **eTable 7.** Event Rates for Recurrent Event Analysis of Buprenorphine Taper Characteristics Associated With OAT Reentry Within 18 Months Postdiscontinuation (N = 6086) **eTable 8.** Recurrent Events Analysis of Buprenorphine Prescribing Characteristics Associated With Return to Prescription Opioids Within 18 Months Posttreatment Discontinuation (N = 6451) **eTable 9. **Sensitivity Analysis of Buprenorphine Taper Characteristics Associated With Opioid Overdose Within 18 Months Postdiscontinuation Among Those With Successful Taper (End Dose ≤ 2 mg) (N = 3154) **eTable 10.** Multivariable Cause-Specific and Subdistribution Hazards Models of Buprenorphine Taper Characteristics Associated With Opioid Overdose Within 18 Months Posttreatment Discontinuation (N = 5774) **eTable 11.** Cause-Specific and Subdistribution Hazards Recurrent Event Analysis of Buprenorphine Taper Characteristics Associated With Opioid Overdose Within 18 Months Posttreatment Discontinuation (N = 6451)
eReferences
