# Supplementary material for: Prescribing Characteristics Associated With Opioid Overdose Following Buprenorphine Taper
Source: JAMA Netw Open. 2022 Sep 29;5(9):e2234168. doi: 10.1001/jamanetworkopen.2022.34168 (PMC9523505; doi:10.1001/jamanetworkopen.2022.34168)
Supplement: Supplement. — eTable 1. Descriptions of All Linked Administrative Databases Used in the Study eTable 2. Covariate Definitions eFigure 1. Study Schematic eAppendix. Calculation of Daily Dose eFigure 2. Cohort Assembly eTable 3. Distribution of the Primary Exposure, Time to Taper Initiation eFigure 3. Kaplan-Meier Curve of Time to Opioid Overdose Following Buprenorphine Taper, Stratified by Time to Taper Start (P = .02) eFigure 4. Kaplan-Meier Curve of Time to Opioid Overdose Following Buprenorphine Taper, Stratified by Milligrams Tapered per Month (P < .001) eFigure 5. Kaplan-Meier Curve of Time to Opioid Overdose Following Buprenorphine Taper, Stratified by Percentage of Days Descending During the Taper Period (P < .001) eFigure 6. Kaplan-Meier Curve of Time to Opioid Overdose Following Buprenorphine Taper, Stratified by Taper Duration (P < .001) eFigure 7. Kaplan-Meier Curve of Time to MOUD Reentry Following Buprenorphine Taper, Stratified by Time to Taper Start (P = .12) eFigure 8. Kaplan-Meier Curve of Time to MOUD Reentry Following Buprenorphine Taper, Stratified by Milligrams Tapered per Month (P < .001) eFigure 9. Kaplan-Meier Curve of Time to MOUD Reentry Following Buprenorphine Taper, Stratified by Percentage of Days the Dose Was Decreasing During the Taper Period (P < .001) eFigure 10. Kaplan-Meier Curve of Time to MOUD Reentry Following Buprenorphine Taper, Stratified by Taper Duration (P < .001) eFigure 11. Kaplan-Meier Curve of Time to Prescription Opioid Use Following Buprenorphine Taper, Stratified by Time to Taper Start (P = .34) eFigure 12. Kaplan-Meier Curve of Time to Prescription Opioid Use Following Buprenorphine Taper, Stratified by Milligrams Tapered per Month (P < .001) eFigure 13. Kaplan-Meier Curve of Time to Prescription Opioid Use Following Buprenorphine Taper, Stratified by Percentage of Days the Dose Was Decreasing During the Taper Period (P = .001) eFigure 14. Kaplan-Meier Curve of Time to Prescription Opioid Use Following Buprenorphine Taper, Strati [file jamanetwopen-e2234168-s001.pdf]

Supplemental Online Content

Bozinoff N, Men S, Kurdyak P, Selby P, Gomes T. Prescribing characteristics associated with opioid overdose following buprenorphine taper. *JAMA Netw Open*. 2022;5(9):e2234168. doi:10.1001/jamanetworkopen.2022.34168

eTable 1. Descriptions of All Linked Administrative Databases Used in the Study
eTable 2. Covariate Definitions
eFigure 1. Study Schematic
eAppendix. Calculation of Daily Dose
eFigure 2. Cohort Assembly
eTable 3. Distribution of the Primary Exposure, Time to Taper Initiation
eFigure 3. Kaplan-Meier Curve of Time to Opioid Overdose Following Buprenorphine Taper, Stratified by Time to Taper Start (*P* = .02)
eFigure 4. Kaplan-Meier Curve of Time to Opioid Overdose Following Buprenorphine Taper, Stratified by Milligrams Tapered per Month (*P* < .001)
eFigure 5. Kaplan-Meier Curve of Time to Opioid Overdose Following Buprenorphine Taper, Stratified by Percentage of Days Descending During the Taper Period (*P* < .001)
eFigure 6. Kaplan-Meier Curve of Time to Opioid Overdose Following Buprenorphine Taper, Stratified by Taper Duration (*P* < .001)
eFigure 7. Kaplan-Meier Curve of Time to MOUD Reentry Following Buprenorphine Taper, Stratified by Time to Taper Start (*P* = .12)
eFigure 8. Kaplan-Meier Curve of Time to MOUD Reentry Following Buprenorphine Taper, Stratified by Milligrams Tapered per Month (*P* < .001)
eFigure 9. Kaplan-Meier Curve of Time to MOUD Reentry Following Buprenorphine Taper, Stratified by Percentage of Days the Dose Was Decreasing During the Taper Period (*P* < .001)
eFigure 10. Kaplan-Meier Curve of Time to MOUD Reentry Following Buprenorphine Taper, Stratified by Taper Duration (*P* < .001)
eFigure 11. Kaplan-Meier Curve of Time to Prescription Opioid Use Following Buprenorphine Taper, Stratified by Time to Taper Start (*P* = .34)
eFigure 12. Kaplan-Meier Curve of Time to Prescription Opioid Use Following Buprenorphine Taper, Stratified by Milligrams Tapered per Month (*P* < .001)
eFigure 13. Kaplan-Meier Curve of Time to Prescription Opioid Use Following Buprenorphine Taper, Stratified by Percentage of Days the Dose Was Decreasing During the Taper Period (*P* = .001)
eFigure 14. Kaplan-Meier Curve of Time to Prescription Opioid Use Following Buprenorphine Taper, Stratified by Taper Duration (*P* < .001)
eTable 4. Event Rates for Buprenorphine Taper Characteristics Associated With Treatment Reentry Within 18 Months Postdiscontinuation (N = 5449)
eTable 5. Recurrent Event Analysis of Buprenorphine Taper Characteristics Associated With Opioid Overdose Within 18 Months Posttreatment Discontinuation (N = 6451)
eTable 6. Recurrent Event Analysis of Buprenorphine Taper Characteristics Associated With Treatment Reentry Within 18 Months Postdiscontinuation (N = 6086)
eTable 7. Event Rates for Recurrent Event Analysis of Buprenorphine Taper Characteristics Associated With OAT Reentry Within 18 Months Postdiscontinuation (N = 6086)

© 2022 Bozinoff N et al. *JAMA Network Open.*

eTable 8. Recurrent Events Analysis of Buprenorphine Prescribing Characteristics Associated With Return to Prescription Opioids Within 18 Months Posttreatment Discontinuation (N = 6451)
eTable 9. Sensitivity Analysis of Buprenorphine Taper Characteristics Associated With Opioid Overdose Within 18 Months Postdiscontinuation Among Those With Successful Taper (End Dose ≤ 2 mg) (N = 3154)
eTable 10. Multivariable Cause-Specific and Subdistribution Hazards Models of Buprenorphine Taper Characteristics Associated With Opioid Overdose Within 18 Months Posttreatment Discontinuation (N = 5774)
eTable 11. Cause-Specific and Subdistribution Hazards Recurrent Event Analysis of Buprenorphine Taper Characteristics Associated With Opioid Overdose Within 18 Months Posttreatment Discontinuation (N = 6451)
eReferences

This supplemental material has been provided by the authors to give readers additional information about their work.

© 2022 Bozinoff N et al. *JAMA Network Open.*

eTable 1. Descriptions of All Linked Administrative Databases Used in the Study

Database	Description
**Narcotics Monitoring System (NMS)**	The Narcotic Monitoring System is a mandatory prescription reporting system in Ontario, which is complete as of July 1, 2012. It captures all outpatient prescriptions for controlled substances in Ontario regardless of payment method. These include all prescription opioids, benzodiazepines (excluding zopiclone), and stimulants[1]. NMS was used to capture all MOUD prescriptions and opioid prescriptions.
**Registered Persons Database (RPDB)**	The RPDB provides demographic information and vital statistics on all persons with an Ontario health card (i.e. OHIP number). We used the RPDB to assess sex, age, and postal code of each individual's primary residence.
**Drug-Alcohol Related Death Database**	DDARD is a dataset of confirmed cause of death from the Office of the Chief Coroner of Ontario and allows the accurate identification of the cause of death. In accordance with Ontario's Coroners Act, all deaths that are sudden and unexpected, or unnatural, must be reported to the Coroner's Office. Opioid-related death was defined as an acute intoxication or toxicity death resulting from the direct contribution of an opioid [2].
**Canadian Institute for Health Information (CIHI) Discharge Abstract Database (DAD)**	Captures details on diagnoses and procedures for all inpatient hospital stays in Ontario. This dataset has been validated through a re-abstraction study which found that the re-abstractor agreed at least in part with the original coder regarding the most responsible diagnosis approximately 85% of the time[3].
**CIHI National Ambulatory Care Reporting System (NACRS)**	Captures details on diagnoses and procedures for all emergency department visits in Ontario.
**CIHI Ontario Mental Health Reporting System (OMHRS)**	The OMHRS database contains demographic, administrative and clinical information for all adult admissions to designated inpatient mental health beds in Ontario. This includes beds in general hospitals as well as in specialty psychiatric facilities. We used OMHRS records to identify psychiatric admissions for the purpose of understanding psychiatric co-morbidity.
**Ontario Health Insurance Plan (OHIP) Database**	This database reflects physician claims through the Ontario Health Insurance Plan. Elements in this dataset include the patient and physician identifiers, the code for service provided, date of service, and associated diagnosis. We used OHIP claims to understand physician specialty, assess healthcare utilization, and in the definition of alcohol use disorder.
**Chronic Obstructive Pulmonary Disease (COPD) Validated Database**	This database uses a validated definition to identify diagnosis dates for all people with COPD in Ontario (85.0% sensitivity, 78.4% specificity). [4]
**HIV Validated Database**	This database uses a validated definition to identify diagnosis dates for all people with HIV in Ontario (96.2% sensitivity, 99.6% specificity).[5]
**Ontario Diabetes Database**	This database uses a validated definition to identify diagnosis dates fo all people with diabetes in Ontario (90.0% sensitivity, 97.7% specificity).[6]
**ICES Physician Database (IPDB)**	IPDB is a dataset that contains yearly information about all physicians in Ontario including demographics, specialty, location and physician activity (billings, workload, types of services provided). For the purpose of our study, IPDB was used to ascertain provider specialty.

© 2022 Bozinoff N et al. *JAMA Network Open.*

eTable 2. Covariate Definitions

Variable	Database	Definition/Citation
Age	RPDB	Age at index using birth date in RPDB
Sex	RPDB	Sex as recorded in RPDB
Rurality of residence	RPDB	Rurality of residence using person's postal code
Neighbourhood income quintile	RPDB	Neighbourhood income quintile using person's postal code
Harmful alcohol use or dependence	OHIP, DAD, NACRS	Physician or hospital related visit for alcohol use disorder using codes below (primary diagnosis) in the 2 years prior to index date:<br>**ICD-10 Codes:**<br>F10 K70 G31.2 G62.1 G72.1 I42.6 K29.2 K86.0 Z50.2 Z71.4 Z86.40 K85.2<br><br>**OHIP Diagnosis Codes:**<br>291 303
Harmful stimulant use or dependence	DAD, NACRS	Hospital visit for harmful stimulant use or dependence using codes below (primary diagnosis) in the 2 years prior to index date:<br>**ICD-10 CA codes:**<br>F140- F159, T436, T405
Harmful sedative-hypnotic use or dependence	DAD, NACRS	Hospital visit for harmful sedative-hypnotic use or dependence using codes below (primary diagnosis) in the 2 years prior to index date:<br>**ICD-10 CA codes:**<br>F131,F132, T423,T424, T426,T427
Hospitalization or ED visit for depression in last 2 years	DAD, NACRS, OMHRS	Hospital visit for depression (primary diagnosis) in the 2 years prior to index date:<br><br>**OMHRS ICD9-CM codes:**<br>DSM5CODE_DISCH1= 296.2x, 296.3x, 296.9x, 300.4x, 311.x, 625.4x. PROVDX_DSM5CODE_ADM1 =4<br><br>**DAD/NACRS ICD-10-CA codes:**<br>F32.x, F33.x, F34.1
Hospitalization or ED visit for anxiety in last 2 years	DAD, NACRS, OMHRS	Hospital visit for anxiety (primary diagnosis) in the 2 years prior to index date:<br><br>**OMHRS ICD9-CM codes:**<br>DSM5CODE_DISCH1 = 293.84, 300, 300.0x, 300.2x, 309.21, 313.23.PROVDX_DSM5CODE_ADM1 = 5

© 2022 Bozinoff N et al. *JAMA Network Open.*

Variable	Database	Definition/Citation
		**<u>DAD/NACRS ICD-10-CA codes:</u>**<br>F064, F40, F41, F93.0-2, F940
John's Hopkins Aggregated Diagnosis Groups (ADGs) [7]	OHIP, DAD	Patients were assigned a weighted score based on presence or absence of 32 ACG® System Aggregated Diagnosis Groups (ADGs) characterizing medical conditions based on their use of inpatient and outpatient health care services in the preceding 2 years [7]. ADGs were summed and used as an ordinal variable.
Emergency Department Visits or hospitalization for opioid poisoning	DAD, NACRS	ED visit or inpatient hospitalization with diagnosis of opioid poisoning using codes below in 1 year prior to index date (admission diagnoses):<br>**<u>ICD-10</u> Codes:**<br>T40.0, T40.1, T40.2, T40.3, T40.4, T40.6
Discipline of prescriber at initiation	OHIP, IPDB	Define pain prescriber as:<br>1)main specialty=anesthesiologist<br>2) physician billed at least 30 x A937 OHIP codes in the 365 days prior to index date
History of methadone in last 6 months	NMS	Any methadone in the 6 months prior to index (excluding index date).
History of buprenorphine in last 6 months	NMS	Any buprenorphine in the 6 months prior to index (excluding index date).
Percentage missed doses	NMS	Number of days during the treatment episode where daily buprenorphine dose=0 divided by total treatment episode in days.
Maximum dose	NMS	Defined as the buprenorphine dose at taper start.
Calendar year of buprenorphine discontinuation	NMS	Buprenorphine treatment episode end date 2013-2015 vs. <u>></u>2016.

ICD-9: International Classification of Diseases, 9th Revision; ICD-10: International Classification of Diseases, 10th Revision, DAD: Discharge Abstract Database, NACRS: National Ambulatory Care Reporting System, OHIP: Ontario Health Insurance Plan, NMS: Narcotics Monitoring System, OMHRS: Ontario Mental Health Reporting System, RPDB: Registered Persons Database.

© 2022 Bozinoff N et al. *JAMA Network Open.*

eFigure 1. Study Schematic

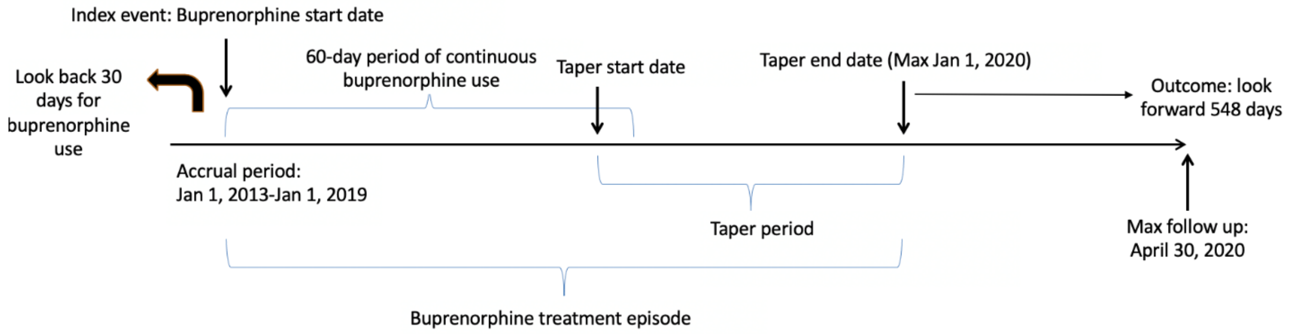

© 2022 Bozinoff N et al. *JAMA Network Open.*

eAppendix. Calculation of Daily Dose

We calculated the buprenorphine dose for every day in the period of continuous buprenorphine use. During the study period, it would have been common for many patients to have daily witnessed ingestion of buprenorphine and for prescribers to give stable clients a maximum of 6 take-home doses per week or 13 take-home doses per 14 days (ie. one witnessed dose per 1-2 weeks) . Prescriptions for buprenorphine in Ontario must include start and stop dates. Buprenorphine prescriptions are typically cancelled if the patient misses 5 or more doses and the pharmacist must contact the prescriber to determine if the dose should be adjusted in these situations, or the patient must see the prescriber to obtain a new prescription[8]. Physicians would generally never prescribe excess supply of buprenorphine, and patients may only pick up their supply on a specified date. Daily dose calculations required data review and cleaning due to variations in clinical practice that exist and anomalies in how witnessed and take-home doses are entered into the NMS.

First, the author undertook a review of a random sample of 30 buprenorphine episodes to identify dispensing patterns and align these with common clinical practice. Following this, the days supplied variable was first corrected to account for instances when doses are witnessed in pharmacy and a take-home supply is dispensed on the same day, and these are entered as separate records within the database. The corrected days supplied was calculated by summing the days supply per person for prescriptions dispensed on the same service date for DINs that represented the same buprenorphine strength. The daily dose was then calculated as corrected quantity dispensed*strength/corrected days supplied. If there were two prescriptions with

© 2022 Bozinoff N et al. *JAMA Network Open.*

different strengths dispensed on the same day with the same days supplied, the daily doses were summed. If a new prescription was issued while there were still days supplied remaining from an old prescription, a daily dose was calculated for the new prescription and assigned as the patient's daily dose moving forward. This would likely reflect the practice of writing a new prescription in the case that buprenorphine take-home doses were lost, but may misclassify rare cases where a dose increase was made by adding additional doses to an existing prescription. If there was no dose for a given day during the episode, the daily dose was zero.

© 2022 Bozinoff N et al. *JAMA Network Open.*

eFigure 2. Cohort Assembly

1

- **Initial cohort**
- Adults >18 with a new buprenorphine prescription between Jan 1, 2013 - Jan 1, 2019 (new is defined by looking back 30 days to ensure no buprenorphine use in last 30 days). Index date is assigned as the date of the first buprenorphine prescription.

2

- **Apply Exclusion Criteria**
- Exclude individuals with missing/invalid IKN, those with missing age or sex, age<18 or age>105, death date prior to index date, individuals who are not an Ontario resident, episodes <60 days.

3

- **Restrict to episodes involving a taper**
- Restrict to episodes where daily dose was ≤2mg in the final dispense of the treatment episode OR where the dose at discontinuation was less than the dose 4 weeks earlier (i.e., dose is decreasing in the last 4 weeks of treatment).
- Exclude episodes where dose never goes above 2 mg.

4

- **Apply exclusions related to data quality**
- Exclude episodes with tapering start dose of >32 mg, where there were >14 days of daily dose=0 in the last 28 days of the episode, episodes with no maximum dose lasting at least 7 consecutive days, and episodes with a taper rate <0.

© 2022 Bozinoff N et al. *JAMA Network Open.*

eTable 3. Distribution of the Primary Exposure, Time to Taper Initiation

Percentile	Days
95	626
90	446
75	249
50 (median)	122
25	72
10	56
5	52

© 2022 Bozinoff N et al. *JAMA Network Open.*

eFigure 3. Kaplan-Meier Curve of Time to Opioid Overdose Following Buprenorphine Taper, Stratified by Time to Taper Start (*P* = .02)

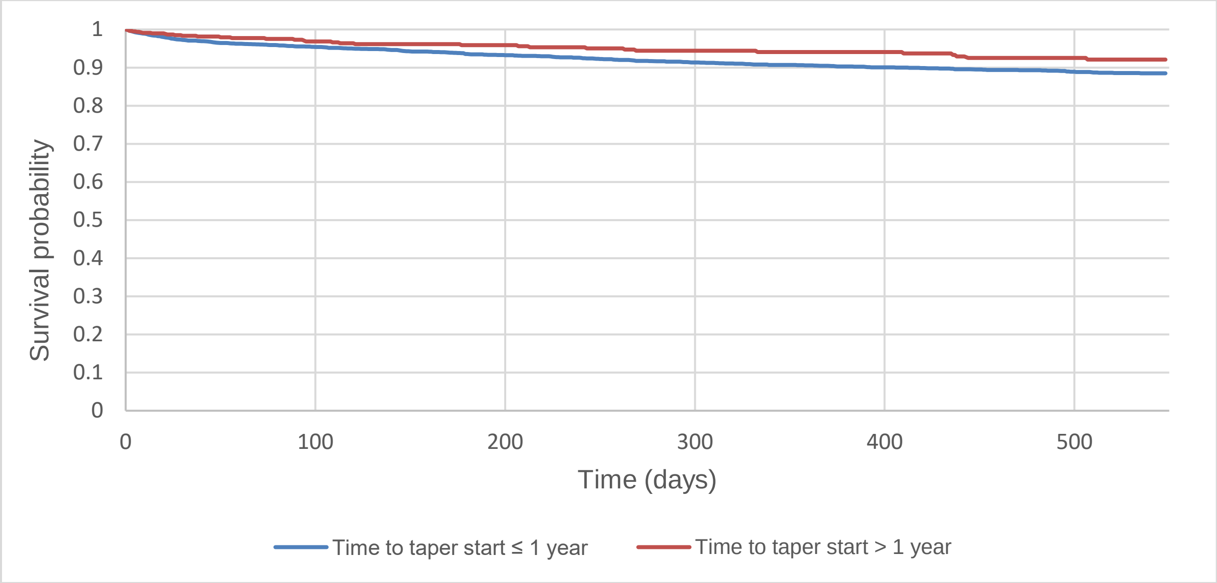

eFigure 4. Kaplan-Meier Curve of Time to Opioid Overdose Following Buprenorphine Taper, Stratified by Milligrams Tapered per Month (*P* < .001)

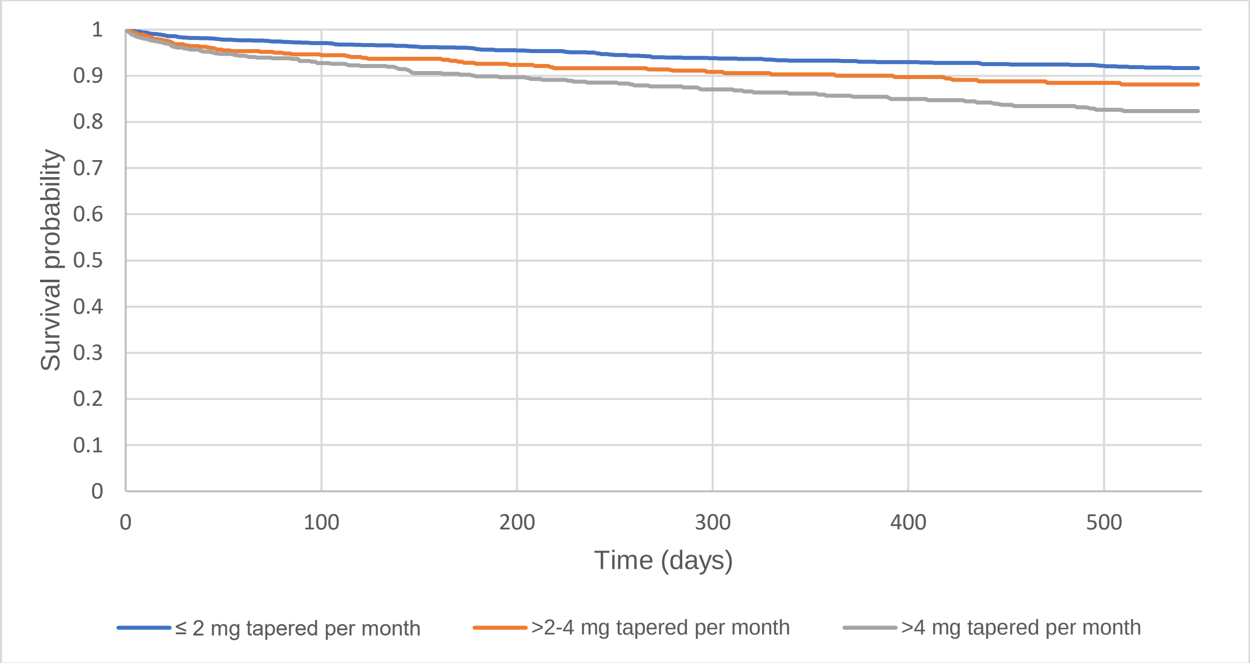

© 2022 Bozinoff N et al. *JAMA Network Open.*

eFigure 5. Kaplan-Meier Curve of Time to Opioid Overdose Following Buprenorphine Taper, Stratified by Percentage of Days Descending During the Taper Period (*P* < .001)

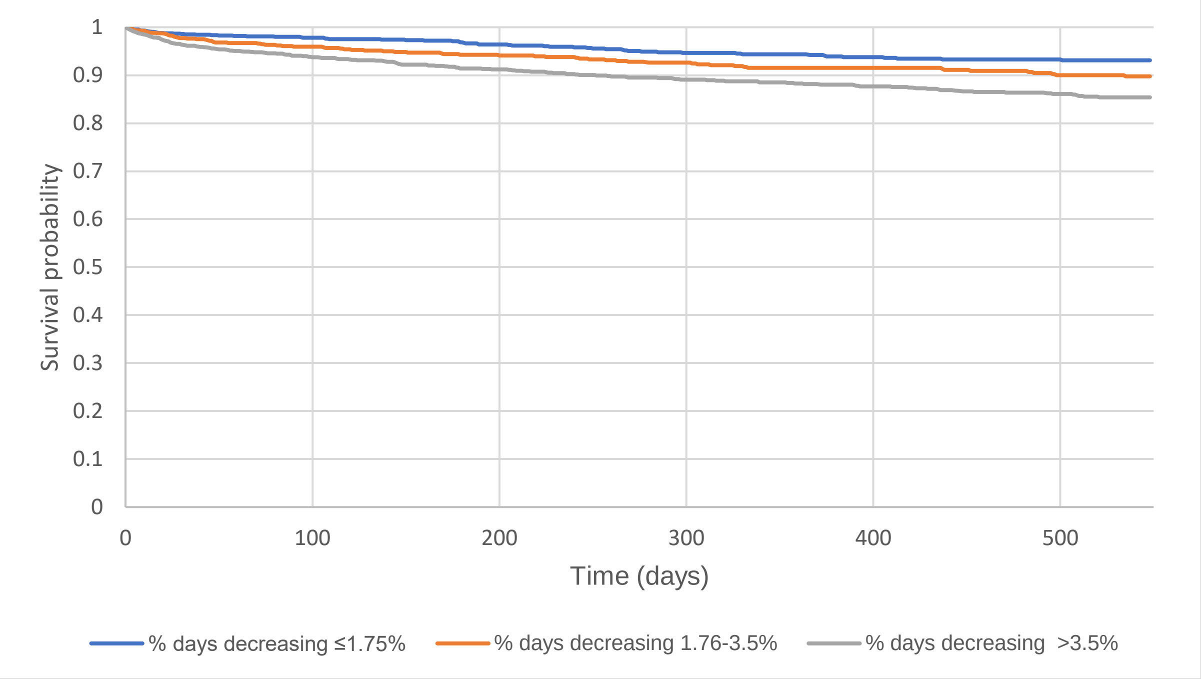

eFigure 6. Kaplan-Meier Curve of Time to Opioid Overdose Following Buprenorphine Taper, Stratified by Taper Duration (*P* < .001)

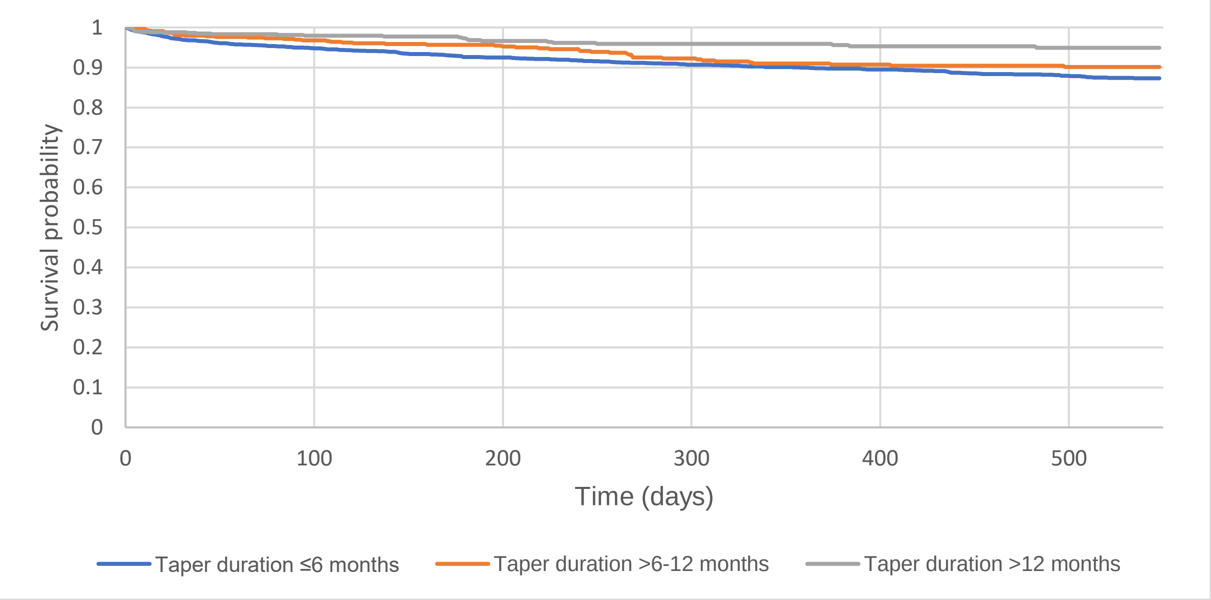

© 2022 Bozinoff N et al. *JAMA Network Open.*

eFigure 7. Kaplan-Meier Curve of Time to MOUD Reentry Following Buprenorphine Taper, Stratified by Time to Taper Start (*P* = .12)

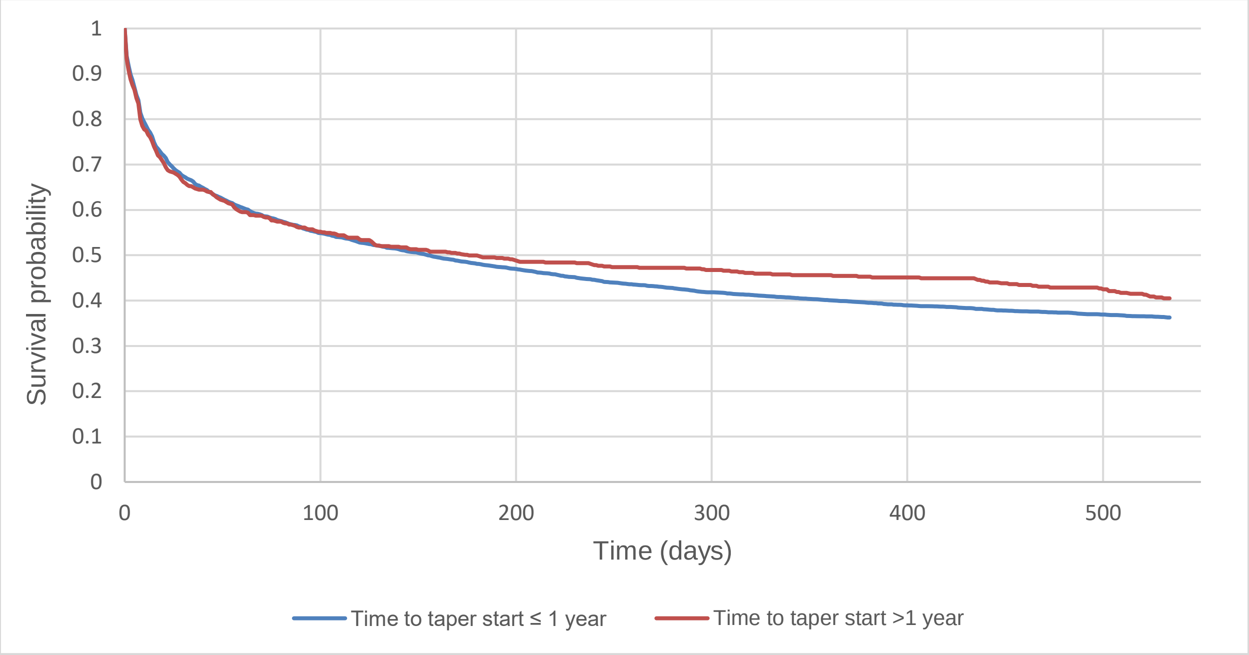

eFigure 8. Kaplan-Meier Curve of Time to MOUD Reentry Following Buprenorphine Taper, Stratified by Milligrams Tapered per Month (*P* < .001)

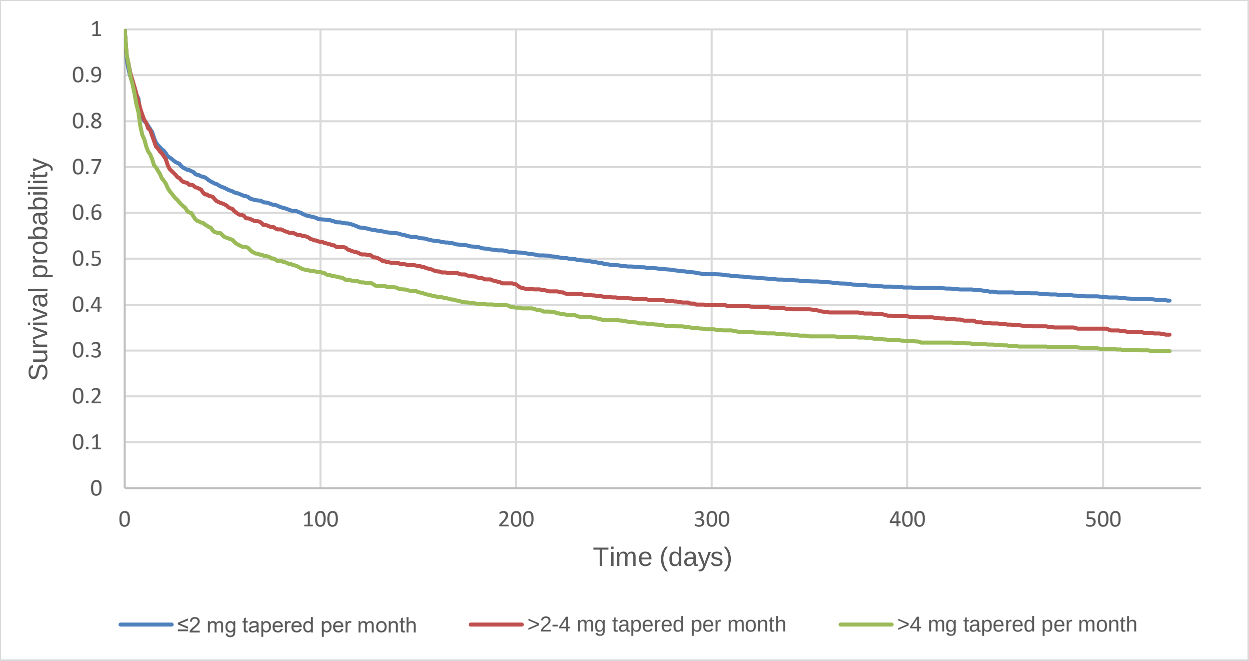

© 2022 Bozinoff N et al. *JAMA Network Open.*

eFigure 9. Kaplan-Meier Curve of Time to MOUD Reentry Following Buprenorphine Taper, Stratified by Percentage of Days the Dose Was Decreasing During the Taper Period (*P* < .001)

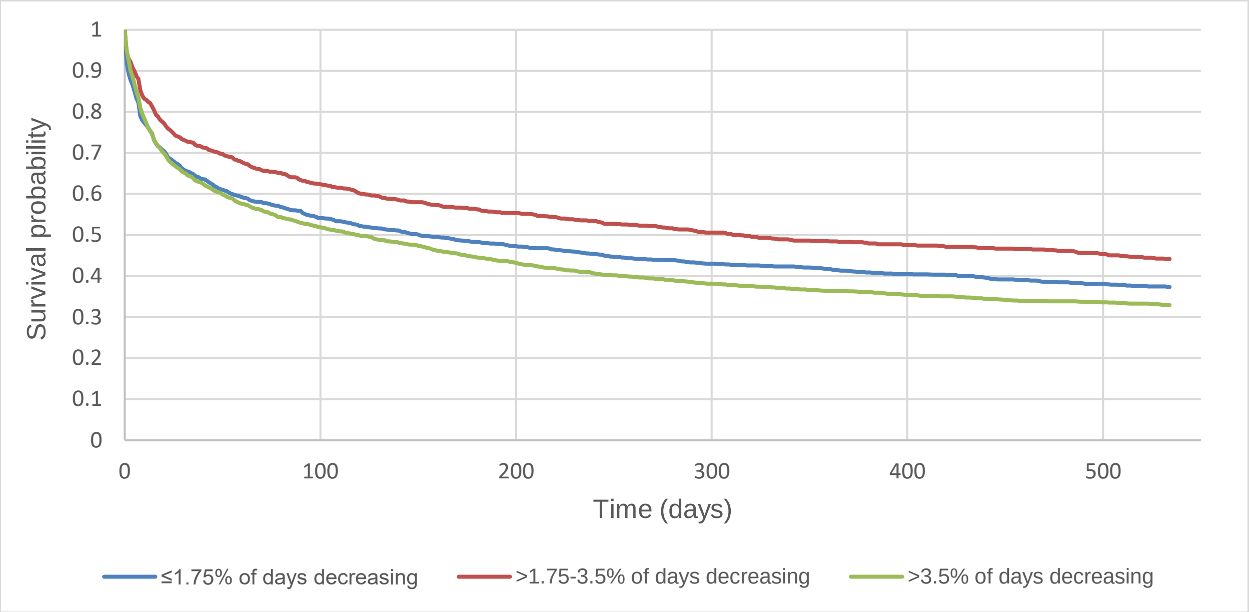

eFigure 10. Kaplan-Meier Curve of Time to MOUD Reentry Following Buprenorphine Taper, Stratified by Taper Duration (*P* < .001)

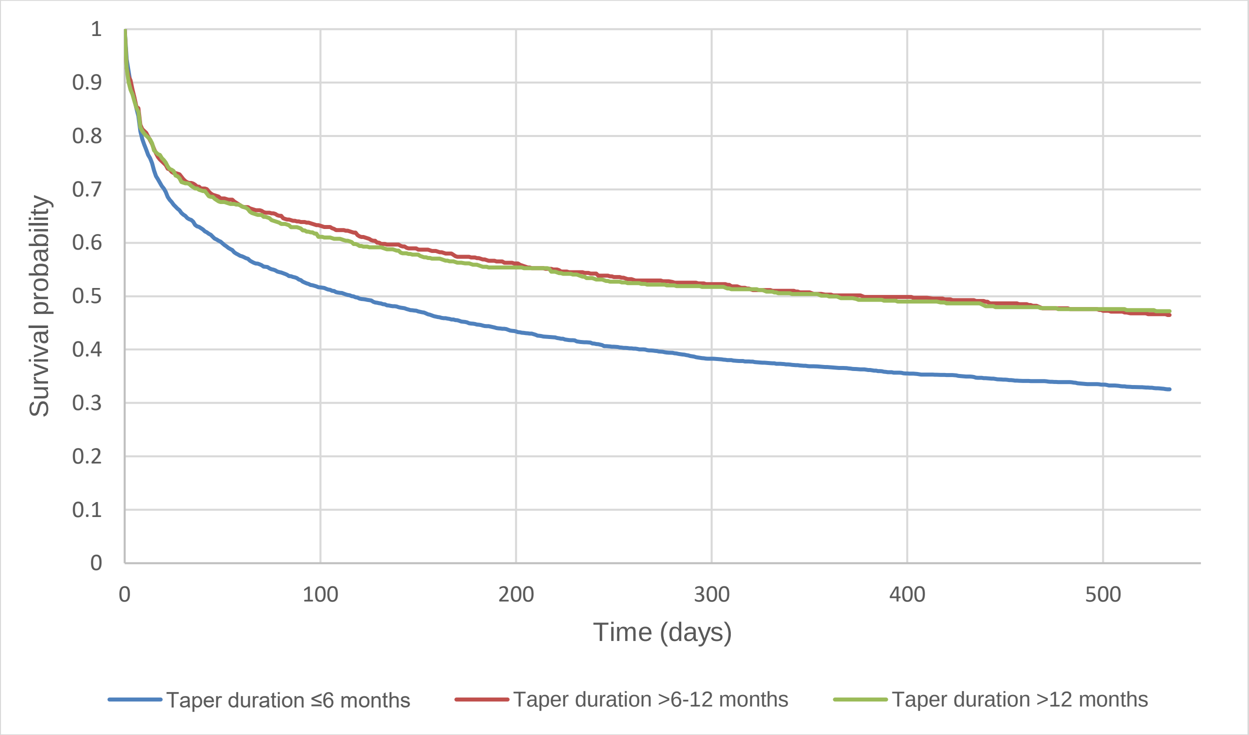

© 2022 Bozinoff N et al. *JAMA Network Open.*

eFigure 11. Kaplan-Meier Curve of Time to Prescription Opioid Use Following Buprenorphine Taper, Stratified by Time to Taper Start (*P* = .34)

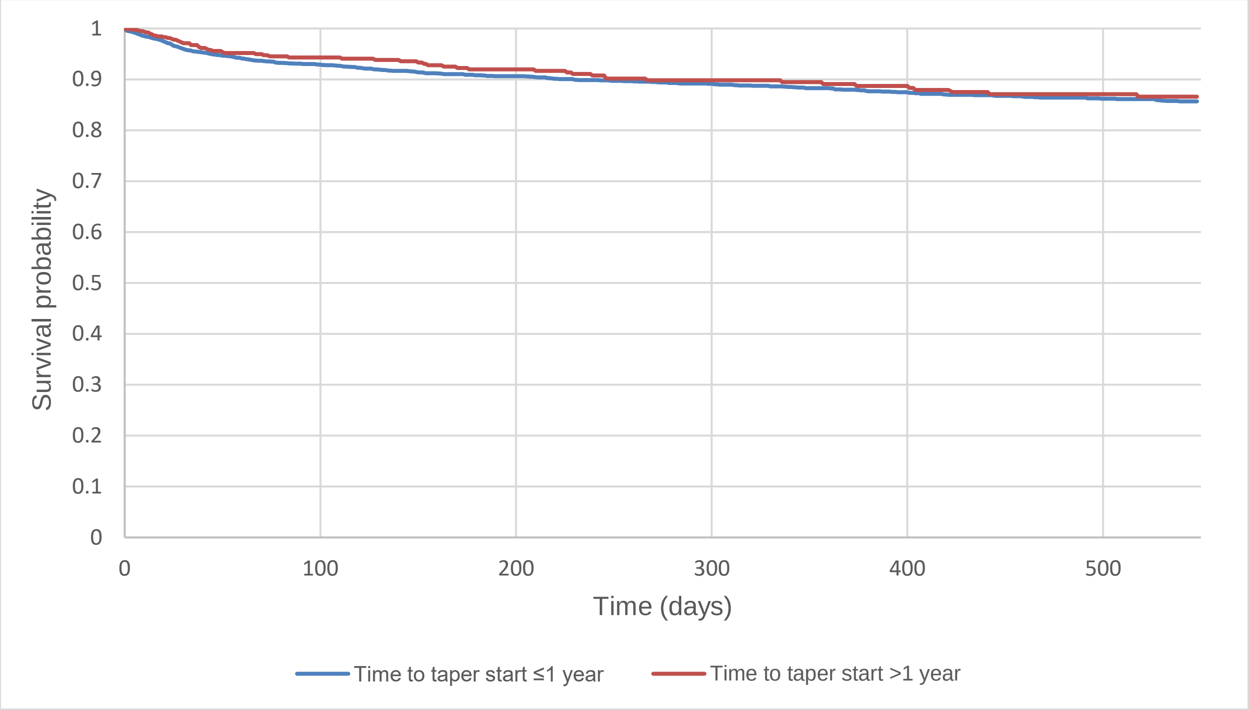

eFigure 12. Kaplan-Meier Curve of Time to Prescription Opioid Use Following Buprenorphine Taper, Stratified by Milligrams Tapered per Month (*P* < .001)

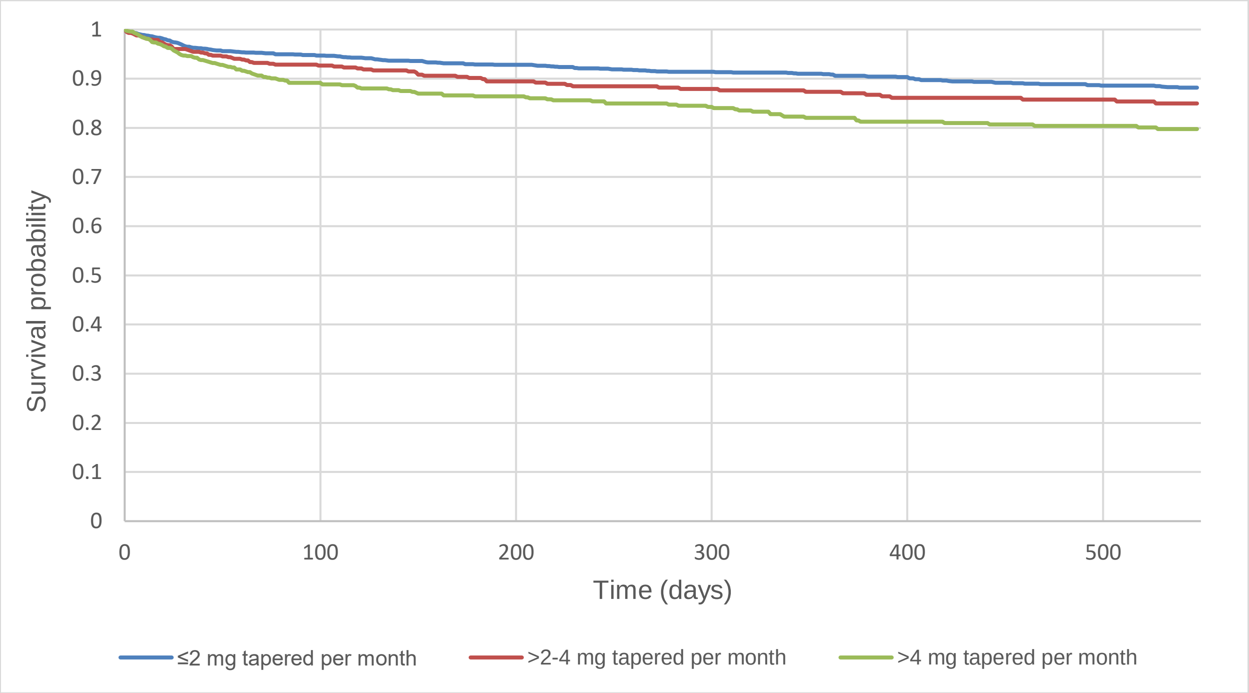

© 2022 Bozinoff N et al. *JAMA Network Open.*

eFigure 13. Kaplan-Meier Curve of Time to Prescription Opioid Use Following Buprenorphine Taper, Stratified by Percentage of Days the Dose Was Decreasing During the Taper Period (*P* = .001)

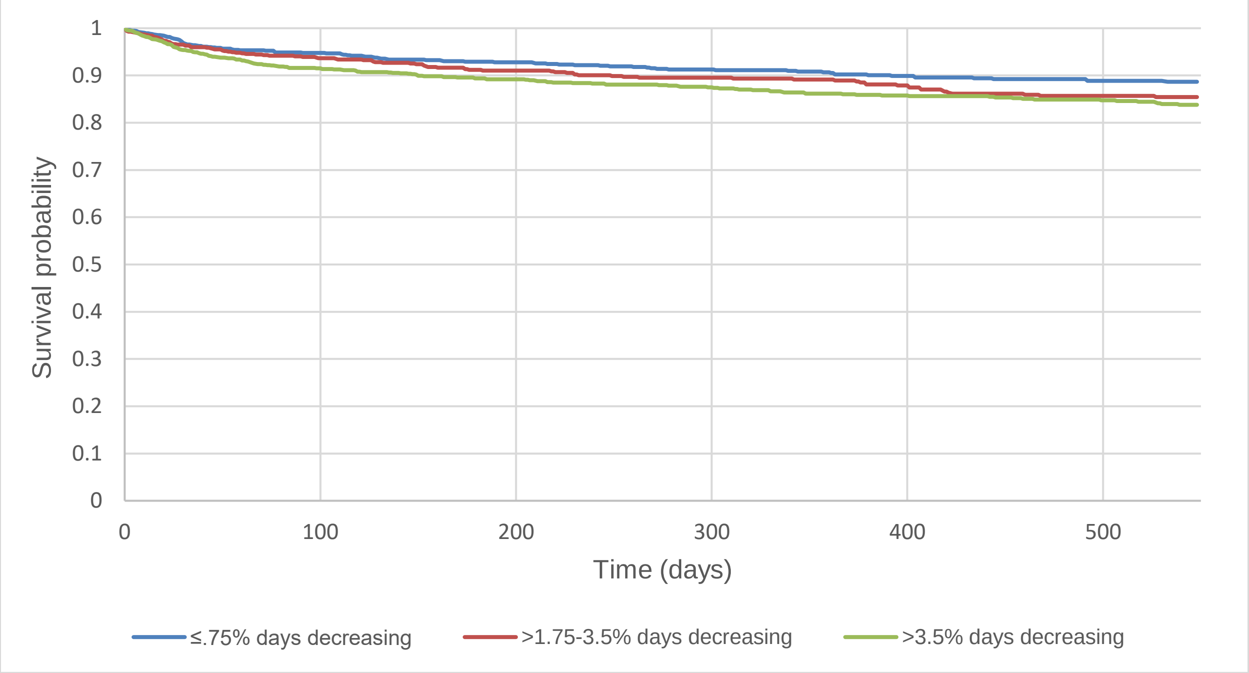

eFigure 14. Kaplan-Meier Curve of Time to Prescription Opioid Use Following Buprenorphine Taper, Stratified by Taper Duration (*P* < .001)

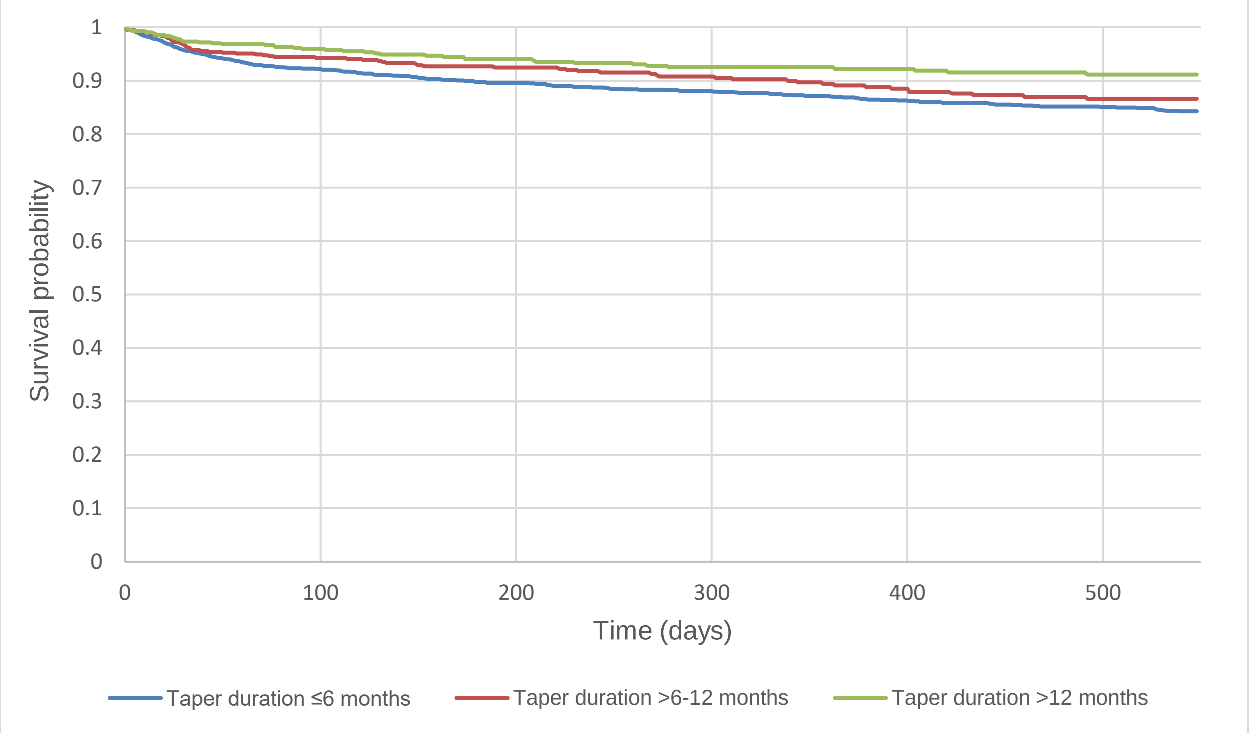

© 2022 Bozinoff N et al. *JAMA Network Open.*

eTable 4. Event Rates for Buprenorphine Taper Characteristics Associated With Treatment Reentry Within 18 Months Postdiscontinuation (N = 5449)

	Total Number of Individuals	Number of individuals with treatment re-entry	Treatment re-entry rate per 100 Person-Years
Time to Taper Initiation			
≤1 year	4680	2921	97.45
>1 year	769	439	90.04
Taper Rate per Month			
≤2 milligrams/month	3147	1811	84.03
>2 to ≤4 milligrams/month	963	626	105.24
>4 milligrams/month	1339	923	125.56
Percentage of days during which dose was decreasing			
≤1.75%	1847	1128	96.53
>1.75 to ≤3.5%	1179	638	73.79
>3.5%	2423	1594	109.79
Taper duration			
≤6 months	3756	2491	109.35
>6 to ≤12 months	865	446	71.08
>1 year	828	423	72.99

© 2022 Bozinoff N et al. *JAMA Network Open.*

eTable 5. Recurrent Event Analysis of Buprenorphine Taper Characteristics Associated With Opioid Overdose Within 18 Months Posttreatment Discontinuation (N = 6451)

	Total Number of Individuals	Number of individuals with opioid overdose	Overdose Rate per 100 Person-Years	Adjusted Hazard Ratio (95% CI)
Time to Taper Initiation				
≤1 year	5544	353	10.56	1.00 [Reference]
>1 year	907	38	6.85	0.69 (0.49-0.98)
Taper Rate per Month				
≤2 milligrams/month	3689	175	7.09	0.63 (0.46-0.87)
>2 to ≤4 milligrams/month	1141	76	11.72	0.70 (0.52-0.93)
>4 milligrams/month	1621	140	17.92	1.00 [Reference]
Percentage of days during which dose was decreasing				
≤1.75%	2165	80	5.93	0.61 (0.42-0.89)
>1.75 to ≤3.5%	1387	86	8.90	0.83 (0.61-1.13)
>3.5%	2899	225	14.22	1.00 [Reference]
Taper duration				
≤6 months	4554	301	11.74	1.00 [Reference]
>6 to ≤12 months	992	58	8.25	1.18 (0.85-1.65)
>1 year	905	32	5.08	0.90 (0.57-1.40)

The proportional hazards assumption was not violated when time-varying covariates were added (p=0.91), nor when examining the martingale residuals.

© 2022 Bozinoff N et al. *JAMA Network Open.*

eTable 6. Recurrent Event Analysis of Buprenorphine Taper Characteristics Associated With Treatment Reentry Within 18 Months Postdiscontinuation (N = 6086)[a]

	**Adjusted Hazard Ratio (95% CI)**		
Time to Taper Initiation			
≤1 year	1.00 [Reference]		
>1 year	0.95 (0.85-1.05)		
*Taper Rate per Month**	182 days	365 days	548 days
≤2 milligrams/month	0.82 (0.72-0.94)	0.95 (0.75-1.19)	1.09 (0.77-1.55)
>2 to ≤4 milligrams/month	1.00 (0.88-1.14)	1.17 (0.91-1.15)	1.38 (0.93-2.05)
>4 milligrams/month	1.00 [Reference]	1.00 [Reference]	1.00 [Reference]
Percentage of days during which dose was decreasing			
≤1.75%	1.27 (1.14-1.42)		
>1.75 to ≤3.5%	0.92 (0.83-1.02)		
>3.5%	1.00 [Reference]		
Taper duration[b]	182 days	365 days	548 days
≤6 months	1.00 [Reference]	1.00 [Reference]	1.00 [Reference]
>6 to ≤12 months	0.69 (0.61-0.80)	0.60 (0.46-0.78)	0.51 (0.34-0.77)
>1 year	0.62 (0.53-0.72)	0.46 (0.34-0.63)	0.34 (0.21-0.55)

[a] Note N=6,086 as outcome window was shifted 14 days in this analysis to account for the fact that discontinuation was defined as no return to buprenorphine within 14 days.

[b] The proportional hazards assumption was violated when time-varying covariates were added ($p<0.001$) and in examining the martingale residuals, therefore Hazard Rates are reported at three times over the follow-up period for taper rate and taper duration.

© 2022 Bozinoff N et al. *JAMA Network Open.*

eTable 7. Event Rates for Recurrent Event Analysis of Buprenorphine Taper Characteristics Associated With OAT Reentry Within 18 Months Postdiscontinuation (N = 6086)

	Total Number of Individuals	Number of individuals with treatment re-entry	Treatment re-entry rate per 100 Person-Years
Time to Taper Initiation			
≤1 year	5235	3316	100.64
>1 year	852	487	90.84
Taper Rate per Month			
≤2 milligrams/month	3558	2074	86.38
>2 to ≤4 milligrams/month	1069	709	110.47
>4 milligrams/month	1459	1020	129.42
Percentage of days during which dose was decreasing			
≤1.75%	2084	1278	97.96
>1.75 to ≤3.5%	1320	730	77.00
>3.5%	2682	1795	113.74
Taper duration			
≤6 months	4258	2867	113.37
>6 to ≤12 months	950	487	70.77
>1 year	878	449	73.15

© 2022 Bozinoff N et al. *JAMA Network Open.*

eTable 8. Recurrent Events Analysis of Buprenorphine Prescribing Characteristics Associated With Return to Prescription Opioids Within 18 Months Posttreatment Discontinuation (N = 6451)

	Total Number of Individuals	Number of individuals with relapse to prescription opioids	Treatment re-entry rate per 100 Person-Years	Adjusted Hazard Ratio (95% CI)
Time to Taper Initiation				
≤1 year	5544	449	14.20	1.00 [Reference]
>1 year	907	68	13.21	0.86 (0.66-1.12)
Taper Rate per Month				
≤2 milligrams/month	3689	261	11.13	0.68 (0.51-0.91)
>2 to ≤4 milligrams/month	1141	98	16.03	0.94 (0.73-1.22)
>4 milligrams/month	1621	158	21.92	1.00 [Reference]
Percentage of days during which dose was decreasing				
≤1.75%	2165	142	11.09	1.02 (0.75-1.38)
>1.75 to ≤3.5%	1387	120	13.21	1.18 (0.90-1.55)
>3.5%	2899	255	17.13	1.00 [Reference]
Taper duration				
≤6 months	4554	385	15.97	1.00 [Reference]
>6 to ≤12 months	992	76	11.46	0.97 (0.74-1.29)
>1 year	905	56	9.27	0.86 (0.61-1.20)

The proportional hazards assumption was not violated when time-varying covariates were added (p=0.14), nor when examining the martingale residuals.

© 2022 Bozinoff N et al. *JAMA Network Open.*

eTable 9. Sensitivity Analysis of Buprenorphine Taper Characteristics Associated With Opioid Overdose Within 18 Months Postdiscontinuation Among Those With Successful Taper (End Dose ≤2 mg) (N = 3154)

	Total Number of Individuals	Number of individuals with opioid overdose	Overdose Rate per 100 Person-Years	Adjusted Hazard Ratio (95% CI)
Time to Taper Initiation				
≤1 year	2740	142	6.89	1.00 [Reference]
>1 year	414	14	4.29	0.63 (0.36-1.11)
Taper Rate per Month				
≤2 milligrams/month	2398	99	5.39	0.56 (0.32-0.96)
>2 to ≤4 milligrams/month	422	25	7.74	0.51 (0.29-0.87)
>4 milligrams/month	334	32	14.03	1.00 [Reference]
Percentage of days during which dose was decreasing				
≤1.75%	1379	38	3.88	0.55 (0.32-0.97)
>1.75 to ≤3.5%	810	45	6.69	0.86 (0.53-1.40)
>3.5%	9651	73	9.95	1.00 [Reference]
Taper duration				
≤6 months	1729	99	7.67	1.00 [Reference]
>6 to ≤12 months	698	39	7.20	1.28 (0.82-2.00)
>1 year	727	18	3.24	0.70 (0.37-1.30)

The proportional hazards assumption was not violated when time-varying covariates were added (p=0.48), nor when examining the martingale residuals.

© 2022 Bozinoff N et al. *JAMA Network Open.*

eTable 10. Multivariable Cause-Specific and Subdistribution Hazards Models of Buprenorphine Taper Characteristics Associated With Opioid Overdose Within 18 Months Posttreatment Discontinuation (N = 5774)

	Total Number of Individuals	Individuals with opioid overdose (N)	Overdose Rate per 100 Person-Years	Adjusted Cause-Specific Hazard Ratio (95% CI)	Adjusted Subdistribution Hazard Ratio (95% CI)
Time to Taper Initiation					
≤1 year	4954	315	10.35	1.00 [Reference]	1.00 [Reference]
>1 year	820	34	6.73	0.69 (0.48-0.997)	0.69 (0.48-1.00)
Taper Rate per Month					
≤2 milligrams/month	3258	154	6.95	0.65 (0.46-0.91)	0.87 (0.63-1.20)
>2 to ≤4 milligrams/month	1026	69	11.48	0.69 (0.51-0.93)	0.82 (0.61-1.10)
>4 milligrams/month	1490	126	17.27	1.00 [Reference]	1.00 [Reference]
Percentage of days during which dose was decreasing					
≤1.75%	1914	71	5.87	0.64 (0.43-0.95)	0.55 (0.37-0.82)
>1.75 to ≤3.5%	1241	76	8.63	0.84 (0.61-1.16)	0.83 (0.61-1.12)
>3.5%	2619	202	13.87	1.00 [Reference]	1.00 [Reference]
Taper duration					
≤6 months	4024	270	11.70	1.00 [Reference]	1.00 [Reference]
>6 to ≤12 months	898	52	8.10	1.12 (0.79-1.60)	1.23 (0.86-1.75)
>1 year	852	27	4.52	0.75 (0.47-1.21)	0.84 (0.51-1.37)

© 2022 Bozinoff N et al. *JAMA Network Open.*

eTable 11. Cause-Specific and Subdistribution Hazards Recurrent Event Analysis of Buprenorphine Taper Characteristics Associated With Opioid Overdose Within 18 Months Posttreatment Discontinuation (N = 6451)

	**Total Number of Individuals**	**Number of individuals with opioid overdose**	**Overdose Rate per 100 Person-Years**	**Cause-Specific Adjusted Hazard Ratio (95% CI)**	**Subdistribution Adjusted Hazard Ratio (95% CI)**
Time to Taper Initiation					
≤1 year	5544	353	10.56	1.00 [Reference]	1.00 [Reference]
>1 year	907	38	6.85	0.69 (0.49-0.98)	0.70 (0.49-0.99)
Taper Rate per Month					
≤2 milligrams/month	3689	175	7.09	0.63 (0.46-0.87)	0.84 (0.62-1.14)
>2 to ≤4 milligrams/month	1141	76	11.72	0.70 (0.52-0.93)	0.81 (0.61-1.08)
>4 milligrams/month	1621	140	17.92	1.00 [Reference]	1.00 [Reference]
Percentage of days during which dose was decreasing					
≤1.75%	2165	80	5.93	0.61 (0.42-0.89)	0.53 (0.37-0.77)
>1.75 to ≤3.5%	1387	86	8.90	0.83 (0.61-1.13)	0.83 (0.62-1.11)
>3.5%	2899	225	14.22	1.00 [Reference]	1.00 [Reference]
Taper duration					
≤6 months	4554	301	11.74	1.00 [Reference]	1.00 [Reference]
>6 to ≤12 months	992	58	8.25	1.18 (0.85-1.65)	1.30 (0.93-1.82)
>1 year	905	32	5.08	0.90 (0.57-1.40)	1.00 (0.63-1.58)

© 2022 Bozinoff N et al. *JAMA Network Open.*

eReferences

1. Monitored Drug List. Ontario Ministry of Health and Long-term Care. Updated March 29, 2021. Accessed April 28, 2021, 2021. https://www.health.gov.on.ca/en/pro/programs/drugs/monitored_productlist.aspx
2. Gomes T, Kitchen SA, Murray R. Measuring the Burden of Opioid-Related Mortality in Ontario, Canada, During the COVID-19 Pandemic. *JAMA Netw Open*. May 3 2021;4(5):e2112865. doi:10.1001/jamanetworkopen.2021.12865
3. Juurlink D, Preyra C, Croxford R, et al. Canadian institute for health information discharge abstract database: a validation study. Institute for Clinical Evaluative Sciences Toronto; 2006.
4. Gershon AS, Wang C, Guan J, Vasilevska-Ristovska J, Cicutto L, To T. Identifying individuals with physcian diagnosed COPD in health administrative databases. *COPD*. 2009:388-94. vol. 5.
5. Antoniou T, Zagorski B, Loutfy MR, Strike C, Glazier RH. Validation of case-finding algorithms derived from administrative data for identifying adults living with human immunodeficiency virus infection. *PLoS One*. 2011;6(6):e21748. doi:10.1371/journal.pone.0021748
6. Lipscombe LL, Hwee J, Webster L, Shah BR, Booth GL, Tu K. Identifying diabetes cases from administrative data: a population-based validation study. *BMC Health Serv Res*. May 2 2018;18(1):316. doi:10.1186/s12913-018-3148-0
7. The John Hopkins University Bloomberg School of Public Health HSRDC. *The John Hopkins ACG® Case-Mix System Version 6.0 Release Notes.* The John Hopkins University 2003.
8. Handford C. Buprenorphine/Naloxone for Opioid Dependence: Clinical Practice Guideline. In: Kahan M, Srivastava A, Cirone S, Sanghera S, Palda V, editors.: Centre for Addiction and Mental Health; 2011.

© 2022 Bozinoff N et al. *JAMA Network Open.*
